# The association of childhood abuse and neglect with tattoos and piercings in the population: evidence from a representative community survey

**DOI:** 10.1186/s40359-022-00811-x

**Published:** 2022-04-22

**Authors:** Mareike Ernst, Ada Borkenhagen, Jörg M. Fegert, Elmar Brähler, Paul L. Plener

**Affiliations:** 1 Department of Psychosomatic Medicine and Psychotherapy, University Medical Center of the Johannes Gutenberg-University Mainz, Untere Zahlbacher Str. 8, 55131 Mainz, Germany; 2 University Hospital for Psychosomatic Medicine and Psychotherapy, University of Magdeburg, Magdeburg, Germany; 3 Department of Child and Adolescent Psychiatry/Psychotherapy, University Hospital Ulm, Ulm, Germany; 4 Research and Treatment Center Adiposity Diseases, Behavioral Medicine Research Unit, Department of Psychosomatic Medicine and Psychotherapy, University Medical Center Leipzig, Leipzig, Germany; 5 Department of Child and Adolescent Psychiatry, Medical University of Vienna, Vienna, Austria

**Keywords:** Adverse childhood experiences, Body modification, Body piercing, Childhood trauma, Population, Tattoo

## Abstract

**Background:**

Tattoos and piercings are becoming increasingly popular in many countries around the world. Individuals seeking such body modifications have reported diverse psychological motives. Besides purely superficial considerations, tattoos and piercings can also have a deep, personal meaning. For example, they can mark and support the emotional processing of significant life events, including formative experiences from early childhood. However, there is a lack of studies that examine the links of tattoos and piercings with experiences of childhood abuse and neglect in large, population-based samples.

**Methods:**

We investigated the association of reports of childhood abuse and neglect with the acquisition of body modifications (tattoos and piercings) within a representative German community sample. Survey participants (*N* = 1060; ages 14–44 years) were questioned whether they had tattoos and piercings and filled out the 28-item Childhood Trauma Questionnaire Short Form (CTQ-SF).

**Results:**

Tattoos and piercings were more common among individuals who reported childhood abuse and neglect. The proportion of participants with tattoos and piercings increased as a function of the severity of all assessed types of abuse and neglect (emotional, physical, and sexual abuse; emotional and physical neglect). In logistic regression analyses which included the covariates age, gender, education, and income, the sum of significant kinds of childhood abuse and neglect was positively related to having tattoos and/or piercings (OR = 1.37 [95% CI 1.19–1.58]).

**Conclusions:**

The results corroborate previous research indicating that body modifications could have special significance for individuals who have survived adversity, in particular interpersonal trauma at the hands of caregivers. These findings could inform screening procedures and low-threshold access to psychotherapeutic care.

## Background

Deliberate body modifications such as tattoos and piercings have a long cultural-historical tradition and are based on techniques that are similar worldwide. Since time immemorial, they have been used as a form of expression, for instance of cultural values, sexual maturity, or of the social status and wealth of the wearer [1]. Long-established tattoo techniques with cultural significance are still present, such as those used by the indigenous peoples of Polynesia or the Inuit, which see the application by hand and simple tools that have hardly changed over hundreds of years. However, modern technological and medical advances have contributed to the proliferation of both tattoos and piercings in today’s society. In many Western countries, they are becoming increasingly popular [2, 3]: Whereas tattoos and piercings used to serve as identifying characteristics of marginalized groups and/or different subcultures [4], they are now a mass phenomenon and reflect a changed attitude towards one’s body: In times of more individualistic lifestyles, the body becomes an aesthetic object which can be actively changed, in accordance with contemporary ideals of self-expression and beauty [5–9]. Tattoos and piercings warrant particular attention as they are usually permanent alterations. Besides health concerns such as allergies and infections [10, 11], they might still imply social sanctioning in some contexts (e.g., at the workplace [12]).

In 2016, 37% of individuals above 14 years who were included in a representative German community study reported having a tattoo. Although tattoos were reported by people of all levels of education and vocational success, they were slightly more common among those with fewer years of school and those currently out of work [13]. Similar proportions of men and women reported having tattoos. By contrast, more women than men reported having piercings (excluding those of the earlobes) [6]. An earlier US-American study had yielded similar results [14].

The underlying psychological motivations for tattoos and piercings have been the focus of comparatively smaller studies, many of which used qualitative methods. Sweetman [7] highlighted that the persistent nature of a tattoo, as well as the involved pain and care, add to its particular significance compared to other fashionable accessories. It is important to note that tattoos and piercings serve as means of communication [15] as they are an outward expression of something felt inwardly. In their review, Wohlrab, Stahl [16] summarized major motivations for acquiring body modifications. These fell into ten categories, comprising superficial motives (such as beauty and fashion) as well as expressions of profound personal meaning (personal narrative, group affiliations and commitment, resistance).

Tattooed and pierced individuals also reported a higher need for uniqueness [17] and lower self-esteem [18] than those without any body modifications. Body modifications have been related to comparatively pronounced risk-taking behavior [19, 20] and sensation seeking [21]. They were more common among individuals with personality disorders [22] and pathological behaviors such as non-suicidal self-injury (NSSI), e.g., in the form of cutting [23, 24].

Along these lines, a recurring theme in the literature has been emotional regulation and coping with stressful life events [25]. In a previous German investigation, participants described the marking of a stage of life, overcoming adversity, and striving to reclaim control over one’s life [26] as motives for the acquisition of piercings and tattoos.

Numerous studies have referred to the importance of previous experiences of bodily harm inflicted by others: In particular survivors of sexual abuse reported the wish to overcome past experiences by means of body modification [27]. An older community study from New Zealand had also found comparatively high rates of childhood sexual abuse among women with tattoos [28]. In a similar way, researchers suggested that a piercing could be an expression of the wish to heal “past wounds” [29]. Piercing may also enable the reconciliation with formerly refused or dissociated body parts [4]. It fits that following periods of healing time promote the occupation with one’s body as well its care [4]. A recent study also found higher rates of childhood neglect and abuse among intimately pierced individuals [30].

However, there is a lack of comprehensive, systematic investigations of the associations of childhood abuse and neglect with tattoos and piercings at the population level. This presents a research gap as adverse childhood experiences are a widespread phenomenon [31], with sustained consequences for health and well-being, identity, and behavior across the life span.

In addition, research has shown that psychological trauma disrupts narrative processing, meaning that memories of adverse events might be represented differently than memories of experiences that were not accompanied by intense distress (see e.g., [32]). This could make it difficult to access and communicate them in verbal form, e.g., in conversation with others. Instead, body modifications lie close as a more physical, behavioral mode of expression.

Furthermore, survivors of childhood abuse and neglect are especially likely to show the characteristics of tattooed and pierced individuals reported above, e.g., low self-esteem, risk-taking and other impulsive behaviors, which are often observed in the context of personality pathology [33, 34]. These factors could facilitate tattoos and piercings in the sense of mediating or moderating variables: As developmental risk factors, abuse and neglect implicate a negative self-image and emotion regulation difficulties (e.g., [35, 36]). Against this background, tattoos and piercings could be used specifically to create more pleasant subjective experiences. This includes feelings of being in control, which contrast the distressing early experience of having been victimized and/or neglected [37]. At the same time, impulsive traits make it more likely that individuals will get (multiple) tattoos or piercings without much concern about potential risks or undesirable long-term consequences, which might otherwise deter them.

The present study:

We used a validated questionnaire assessing childhood abuse and neglect, the 28-item short form of the Childhood Trauma Questionnaire (CTQ-SF) [38], in a representative population sample. We presumed that childhood abuse and neglect are consequential early life experiences that are positively associated with body modifications later in life, e.g., based on previous evidence from survivors of sexual abuse [27, 28] and individuals with intimate piercings [30]. We thus expected higher rates of tattoos and piercing among individuals reporting abuse and neglect compared to those reporting no abuse or neglect. We also expected reports of more severe abuse and neglect to be associated with higher proportions of tattoos and piercings among the persons affected.

Tattoos and piercings are in some respects comparable (e.g., both are permanent and the experience of getting them is painful to some degree), however, piercing the skin versus applying an image or lettering to it are different kinds of body modifications. Therefore, given the lack of studies that have systematically investigated associations of (childhood) adversity with tattoos and piercings within the same sample, more exploratory research questions concerned potentially differential associations of childhood abuse and neglect with tattoos versus with piercings.

Further, as women are more likely to experience childhood abuse and neglect [39], it is an open question whether the association of childhood abuse and neglect and piercings in particular remains robust if gender differences are statistically controlled.

### Methods

#### Survey Strategy

A representative sample of the German population was surveyed by the independent demographic consulting company USUMA (based in Berlin, Germany) from 09/2016 to 11/2016. Participants were chosen via random-route procedure. All participants were at least 14 years of age and had sufficient understanding of the German language. They were informed of the study procedures, data collection, and anonymization of personal data before providing informed consent. In the case of minors, participants gave informed assent with informed consent being provided by their parents/legal guardians. The sample was representative of the German population with respect to age, gender, and level of education. Out of 4902 designated addresses, 2510 households participated. Individuals in multi-person households were randomly selected using a Kish-Selection-Grid. Responses were anonymous. Socio-demographic information was obtained in a face-to-face interview conducted by trained interviewers. All other information was gathered in written form (pen and paper) as part of a questionnaire that was handed out together with a sealable envelope. It included questions about tattoos and piercings and the 28-item Childhood Trauma Questionnaire Short Form. The study was conducted in accordance with the Declaration of Helsinki and fulfilled the ethical guidelines of the International Code of Marketing and Social Research Practice of the International Chamber of Commerce and of the European Society of Opinion and Marketing Research. The study materials and procedure were approved by the Ethics Committee of the Medical Department of the University of Leipzig (number 297/16ek).

In order to establish comparability with previous studies investigating tattoos and piercings in the German population [40] and to focus on a younger age group in which body modification is of higher relevance, we only included participants aged 14–44 years (reducing the sample to *N* = 1060).

### Measures

#### Sociodemographic information

Participants reported their age, gender, and educational attainment. We calculated equivalised income according to the OECD guideline [41] by dividing the household income through the square root of people in household. The result was then recoded into the following categories: 1 ≤ 1250€, 2 = 1250–2500€, 3 ≥ 2500€.

#### Tattoos and piercings

The presence of tattoos and piercings was assessed via self-report. The questions were “Do you have tattoos?” and “Do you have piercings (not including those of the earlobes)?”. Response options were “No”, “Yes, one”, and “Yes, multiple”.

#### Childhood abuse and neglect

Experiences of abuse and neglect were assessed using the 28-item short form of the Childhood Trauma Questionnaire (CTQ-SF) [38]. It comprises five subscales: emotional abuse, physical abuse, sexual abuse, emotional neglect, and physical neglect. Each of the 28 items (e.g., “I had to wear dirty clothes”, assessing physical neglect) is scored on a five-point Likert scale (ranging from 1 = never to 5 = very often). Responses to the single items are then summarized. For each subscale, the sum score ranges from 5 to 25 points. The total score of the questionnaire is the sum of the five subscales. The CTQ-SF has been widely used in community samples as well as in clinical practice and research. Klinitzke, Romppel [39] confirmed its five-factor-structure and attested to the scales’ acceptable to good internal consistencies (Cronbach’s α = 0.62–0.96). We also confirmed acceptable to good internal consistencies based on the present sample (emotional abuse: ω = 0.83, physical abuse: ω = 0.78, sexual abuse: ω = 0.86, emotional neglect: ω = 0.87, and physical neglect: ω = 0.65).

### Statistical procedure

In this study, the coding of the severity (none to minimal, low to moderate, moderate to severe, severe to extreme) of the five different kinds of childhood abuse and neglect assessed by the CTQ-SF followed established, widely used norms. These were based on previous representative surveys of the German population [42]. For example, for the subscale emotional abuse, none to minimal ranges from 5 to 8 points, low to moderate from 9 to 12 points, moderate to severe from 13 to 15 points, and severe to extreme from 16 to 25 points.

In line with this previous investigation, the categories were also combined into “non-significant” (including only none to minimal abuse/neglect) and “significant” reports (combining the three categories low to moderate, moderate to severe, and severe to extreme).

In order to control for potential confounders of the associations of interest, we calculated multivariate logistic regression models of the presence of body modifications (including separate analyses of the presence of tattoos and piercings). These models included participants’ age (as a continuous variable), gender (coded 1 = men, 2 = women), equivalized household income, level of education (1 = lower than the German Abitur, 2 = (comparable to the) German Abitur or higher), and the sum of “significant” kinds of abuse and neglect (referring to the five subscales of the CTQ-SF, using the cut-offs detailed above) as a continuous variable.

P-values correspond to two-tailed tests. Confidence intervals (CIs) are reported for Odds Ratios (OR). Analyses were carried out using R Version 4.0.3. We calculated the phi coefficient (φ) for associations of dichotomous variables, i.e., comparisons of proportions via *χ*^*2*^-tests, and Cohen’s *d* as an effect size measure for standardized differences of mean values, i.e., comparisons conducted via t-tests. Effect sizes and regression coefficients are interpreted following Cohen[43]. Due to the small amounts of missing data (< 2% per variable), we used list-wise deletion.

## Results

### Participants

We analyzed data of 1060 participants. This sample comprised 560 women (52.8%). Participants’ mean age was 30.47 years (*SD* = 8.41). Roughly a fifth of participants had the German Abitur (general university admission, usually obtained after 12–13 years of school) (*N* = 282, 26.6%), and most participants’ income fell into the lowest income bracket (*N* = 628, 59.2%).

### Prevalence of tattoos and piercings

In total, 38.1% (*N* = 404) of the sample reported to have at least one tattoo or piercing. Tattoos were more common (*N* = 339, 32.0%) than piercings (*N* = 212, 20.0%). Comparable proportions of men and women reported having tattoos, while piercings were more common among women [*χ*^2^(1, *N* = 1058) = 42.52, *p* < 0.001, φ = 0.20]. Having multiple tattoos was also similarly common among men and women, but more women than men reported having multiple piercings [*χ*^2^(1, *N* = 1060) = 16.80, *p* < 0.001, φ = 0.13]. There were 143 participants (13.5%) who reported to have both (at least one tattoo and at least one piercing).

### Prevalence of childhood abuse and neglect

On the basis of the cut-offs established by Häuser, Schmutzer [42], at least one kind of “significant” childhood abuse or neglect was reported by 24.6% of participants (*N* = 261). Physical forms of abuse or neglect were reported by more participants (*N* = 223, 21.0%) than emotional forms (*N* = 155, 14.6%). Those reporting childhood abuse or neglect were more likely to be women.

(*χ*^*2*^(1, *N* = 1060) = 9.29, *p* = 0.003, φ = 0.09) and to have a lower level of education the German Abitur (*χ*^*2*^(1, *N* = 1058) = 5.69, *p* = 0.019, φ = 0.07). They were also older (*M* = 31.48, *SD* = 7.99) than those without childhood abuse and neglect (*M* = 30.14, *SD* = 8.54) (*t*(1054) = 2.24, *p* = 0.025, *d* = 0.16). No differences were observed with respect to income.

### Association of childhood abuse and neglect with tattoos and piercings

**Fig. 1:**
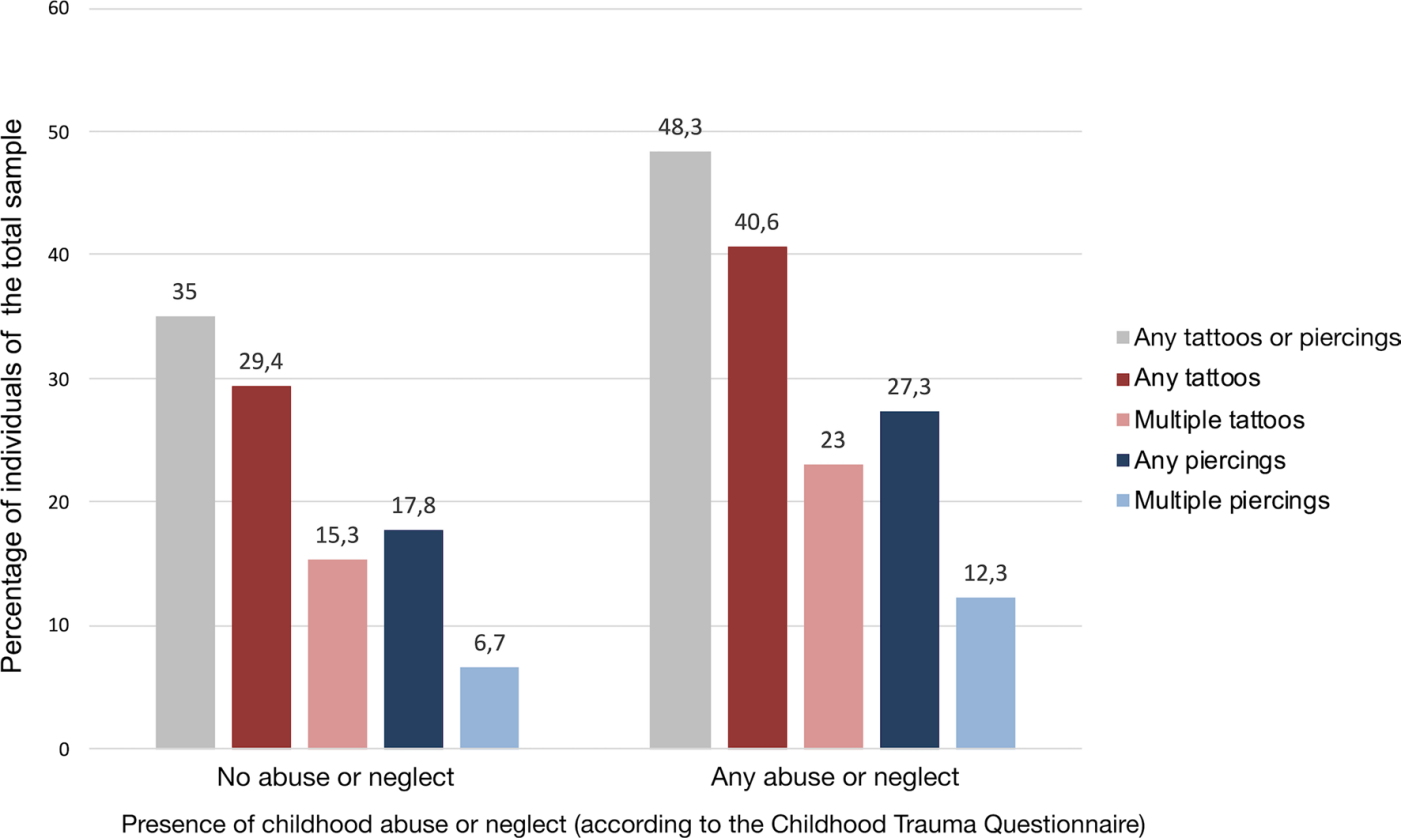
Percentage of individuals with tattoos and piercings, stratified by reports of childhood abuse and neglect. Proportions of those with tattoos or piercings (or several tattoos or piercings, respectively), were greater among those who reported adverse childhood experiences. All presented differences between those without childhood adversity and those with reports of childhood adversity were statistically significant

Overall, 48.3% of those reporting at least one kind of abuse or neglect also reported to have at least one tattoo or piercing, compared to 35% among those who reported no childhood abuse or neglect. This difference was statistically significant (*χ*^*2*^(1, *N* = 1058) = 14.45, *p* < 0.001, φ = 0.12). Likewise, 40.6% of participants who reported at least one kind of abuse or neglect had at least one tattoo, compared to 29.4% of those who did not report any “significant” abuse or neglect (*χ*^*2*^(1, *N* = 1058) = 11.35, *p* = 0.001, φ = 0.11). Similar ratios were observed regarding piercings: 27.3% of individuals who reported abuse or neglect also reported at least one piercing, compared to 17.8% of those who did not report abuse or neglect (χ^2^(1, *N* = 1058) = 10.86, *p* = 0.001, φ = 0.10). Group differences were similar for participants reporting multiple tattoos or piercings (see Fig. 1).

#### Differentiation by severity and kind of abuse and neglect

**Fig. 2:**
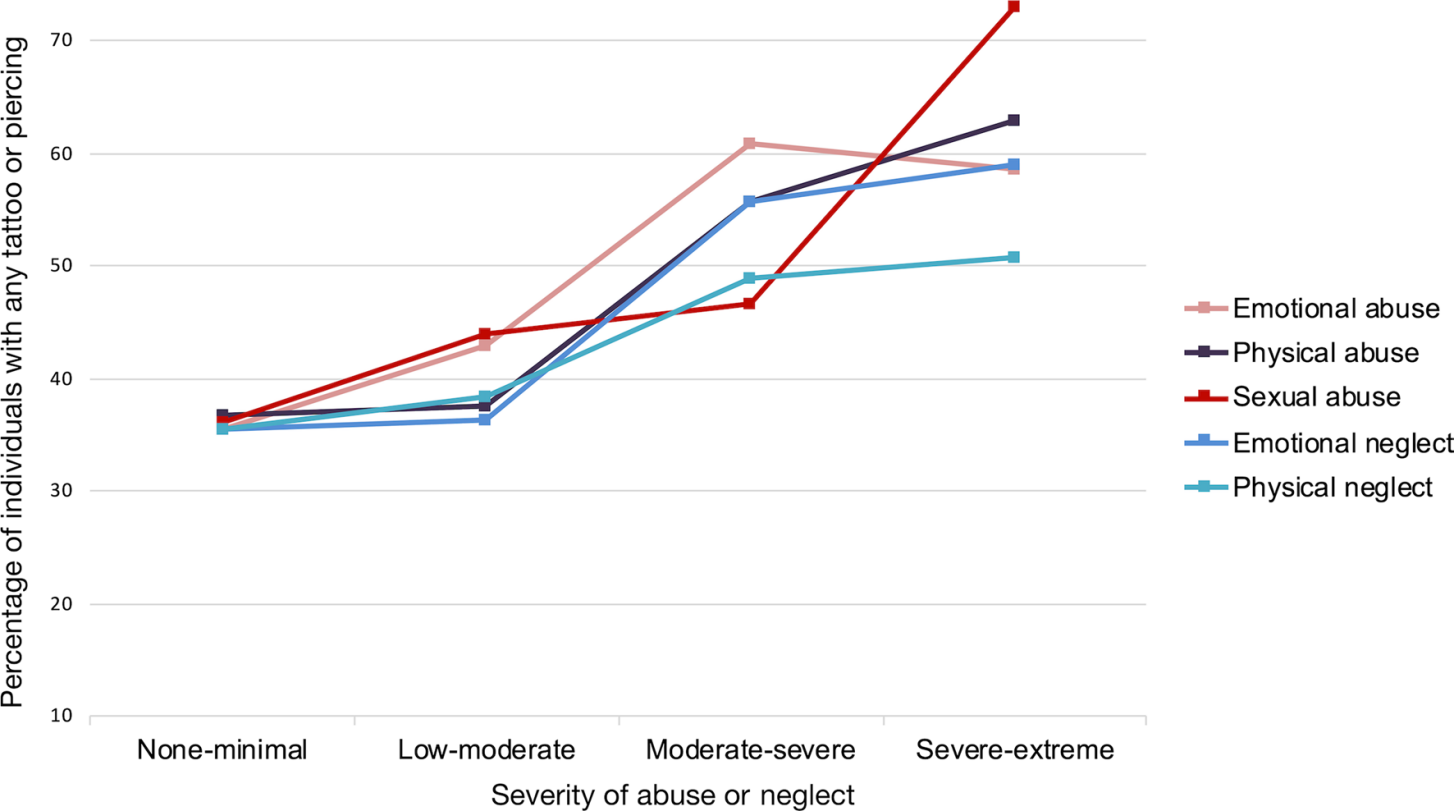
Percentage of individuals with any tattoo or piercing among participants reporting different degrees of the five types of childhood abuse and neglect. More severe forms of abuse and neglect were associated with more reports of at least one tattoo or piercing

**Fig. 3:**
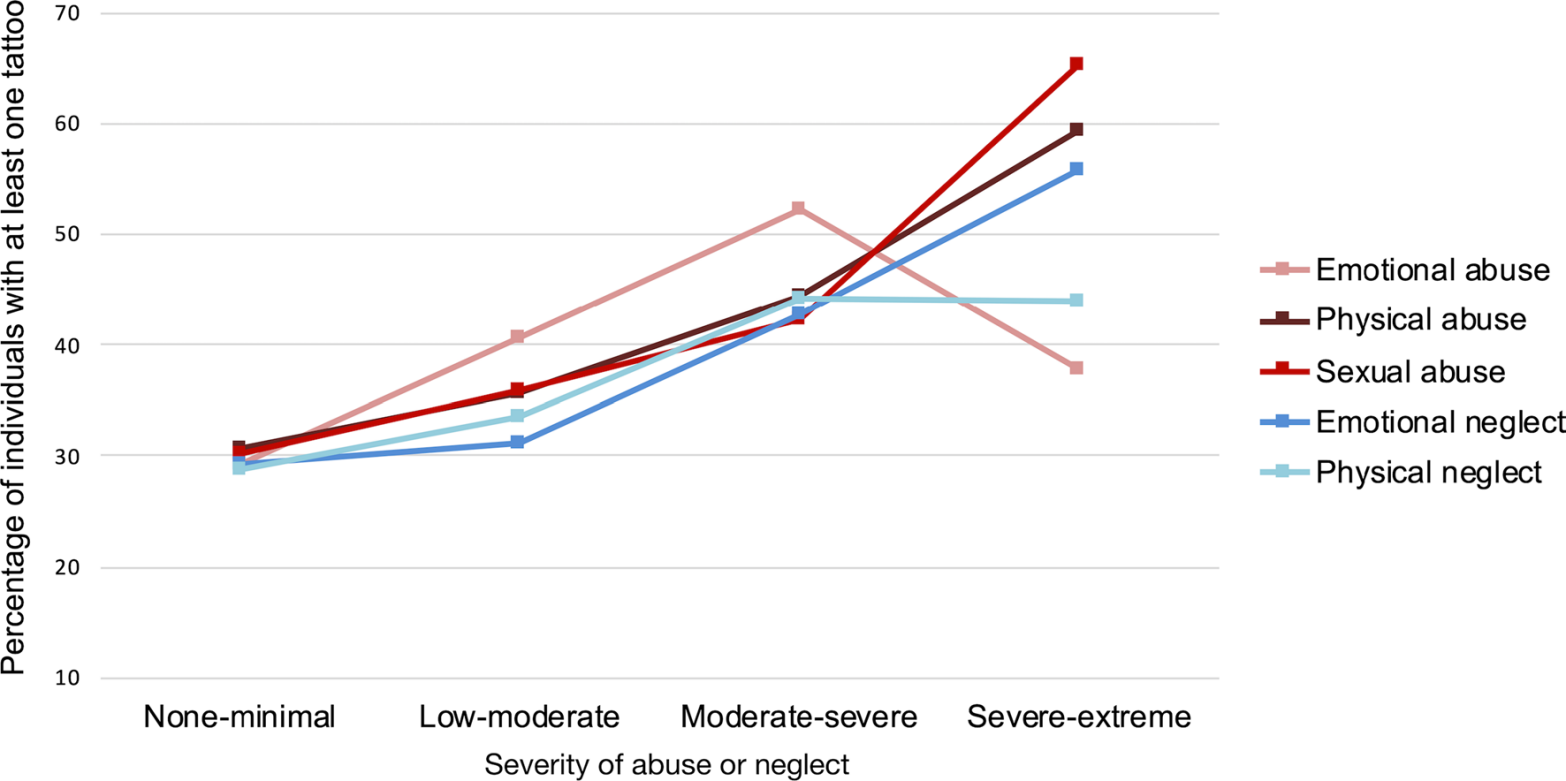
Percentage of individuals with at least one tattoo among participants reporting different degrees of the five types of childhood abuse and neglect. The percentage of persons with tattoos increased as a function of more severe abuse and neglect

**Fig. 4:**
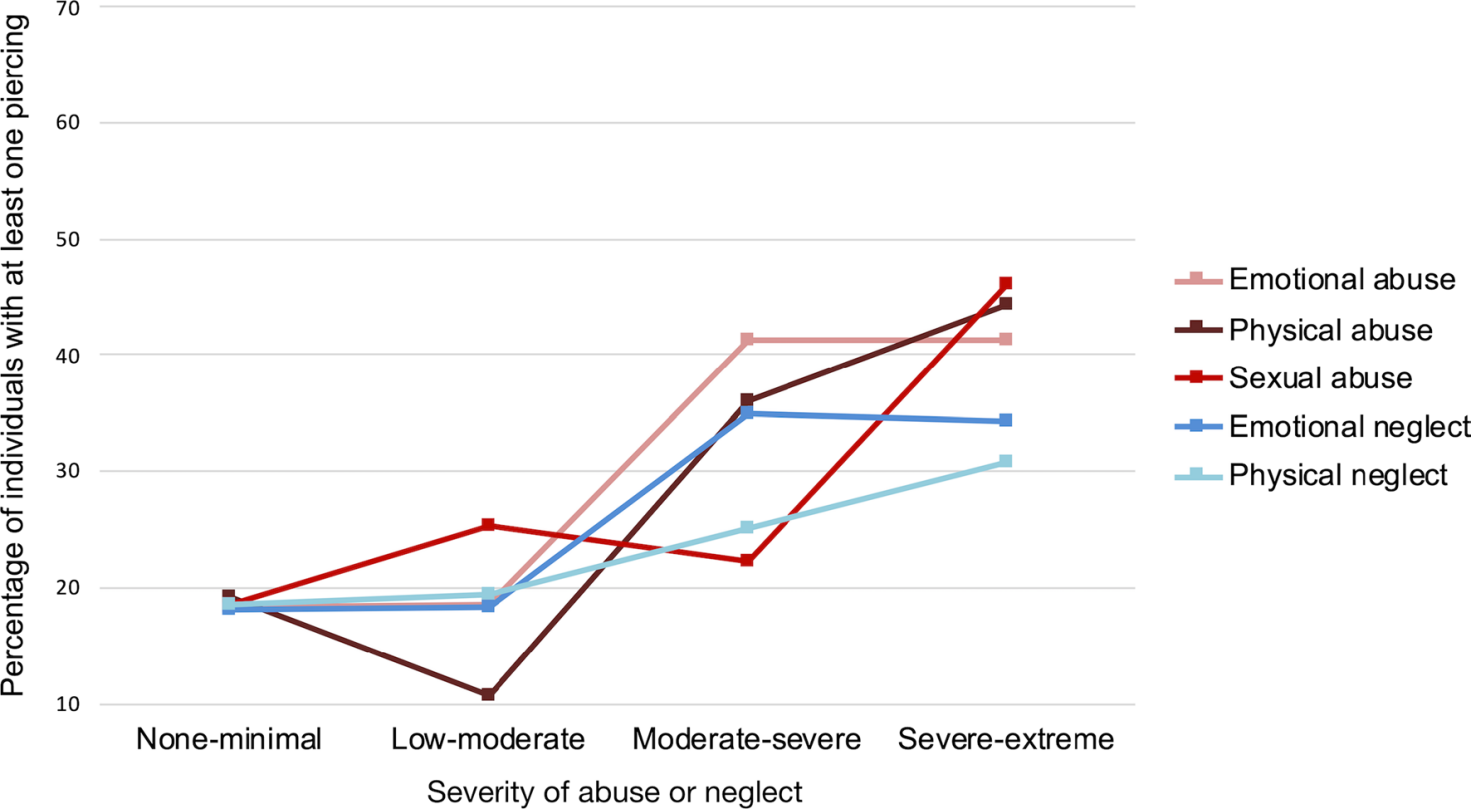
Percentage of individuals with at least one piercing among the groups of participants reporting different degrees of the five types of childhood abuse and neglect. The percentage of persons reporting tattoos increased as a function of more severe abuse and neglect

We tested whether the presence of any tattoo or piercing was related to the severity of the different kinds of abuse or neglect. We found significant effects of the severity of emotional abuse (*χ*^*2*^(3, *N* = 1057) = 18.74, *p* < 0.001, φ = 0.13), physical abuse (*χ*^*2*^(3, *N* = 1057) = 12.10, *p* = 0.007, φ = 0.11), sexual abuse (*χ*^*2*^(3, *N* = 1057) = 17.06, *p* = 0.001, φ = 0.13), emotional neglect (*χ*^*2*^(3, *N* = 1057) = 21.28, *p* < 0.001, φ = 0.14) and physical neglect (*χ*^*2*^(1, *N* = 1057) = 11.21, *p* = 0.011, φ = 0.10). Figure 2 shows the proportion of participants with any tattoo or piercing (stratified by the severity of different kinds of abuse and neglect). Figures 2, 3 and 4 all depict the same range on the y-axis (10–75%) so that relative differences can be visually inferred.

We also investigated tattoos and piercings separately. The proportion of individuals with tattoos varied significantly as a function of the severity of all assessed kinds of childhood abuse and neglect (emotional abuse: *χ*^*2*^(3, *N* = 1057) = 16.18, *p* = 0.001, φ = 0.12; physical abuse: *χ*^*2*^(3, *N* = 1057) = 12.87, *p* = 0.005, φ = 0.11; sexual abuse (*χ*^*2*^(3, *N* = 1057) = 17.29, *p* = 0.001, φ = 0.13; emotional neglect: *χ*^*2*^(3, *N* = 1057) = 21.54, *p* < 0.001, φ = 0.14; and physical neglect: *χ*^*2*^(3, *N* = 1057) = 14.43, *p* = 0.002, φ = 0.12). These results are visualized in Fig. 3.

Regarding piercings, similar effects of severity were found with respect to four CTQ-SF subscales: Emotional abuse (*χ*^*2*^(3, *N* = 1057) = 22.69, *p* < 0.001, φ = 0.15), physical abuse (*χ*^*2*^(3, *N* = 1057) = 19.16, *p* < 0.001, φ = 0.14), sexual abuse (*χ*^*2*^(3, *N* = 1057) = 13.59, *p* = 0.004, φ = 0.11), and emotional neglect (*χ*^*2*^(3, *N* = 1057) = 18.46, *p* < 0.001, φ = 0.13). However, this was not the case for physical neglect (*p* = 0.054). The association of the presence of piercings with severity of abuse and neglect is shown in Fig. 4.

#### Regression analyses

As both the exposure to childhood abuse and neglect and the presence of tattoos and piercings varied depending on participants’ socio-demographic characteristics, we investigated associations of body modifications and childhood abuse and neglect in multivariate analyses which included these potential confounders as covariates (Table 1). There was still a positive association of the number of “significant” kinds of abuse and neglect and the likelihood to report any tattoos or piercings (OR = 1.37 (95% CI 1.19–1.58)). The effect applied to tattoos (OR = 1.34 (95% CI 1.16–2.54)) and to piercings (OR = 1.30 (95% CI 1.12–1.51)).

**Table 1:** Logistic regression analyses of having any body modification, at least one tattoo, or at least one piercing on socio-demographic characteristics and childhood abuse and neglect

	Any tattoo or piercing (*N* = 404)^1^			At least one tattoo (*N* = 339)^2^			At least one piercing (*N* = 212)^3^		
	OR	95% CI (L, U)	*p*	OR	95% CI (L, U)	*p*	OR	95% CI (L, U)	*p*
Female gender	1.45	1.11; 1.89	0.007	1.11	0.84; 1.46	0.47	3.20	2.23; 4.59	< 0.001
Age	1.00	0.98; 1.01	0.66	1.01	0.99; 1.02	0.54	0.98	0.96; 1.00	0.020
Income	1.09	0.85; 1.40	0.49	1.02	0.79; 1.32	0.89	1.35	0.99; 1.84	0.062
Education	0.68	0.50; 0.92	0.014	0.54	0.39; 0.76	< 0.001	0.95	0.65; 1.39	0.79
Sum of "significant” kinds of abuse and neglect	1.37	1.19; 1.58	< 0.001	1.34	1.16; 1.54	< 0.001	1.30	1.12; 1.51	0.001

^1^Nagelkerke R^2^ = 0.053; ^2^Nagelkerke R^2^ = 0.051; ^3^Nagelkerke R^2^ = 0.098

## Discussion

This study used a validated assessment of childhood adversity in a representative sample of the German population. We found consistent associations of abuse and neglect and the presence of body modifications. Not only were tattoos and piercings more common among those who reported any kind of childhood adversity, their prevalence rates also increased with greater severity of all kinds of abuse and neglect.

Thus, the results complement previous studies which focused on specific (risk) groups [27, 30, 44], as individuals with body modifications in our study were part of a random sample. They had not been recruited because of these characteristics and/or the special relevance their tattoos and piercings had for them, personally. The socio-demographic differences among participants with and without tattoos and piercings corresponded to prior representative investigations in the German context [6, 13]. However, we also observed a positive association of the sum of significant kinds of childhood adversity and tattoos and piercings in multiple logistic regression analyses that statistically controlled the effects of variables such as age and level of education. Given the growing popularity of tattoos and piercings among younger individuals due to their aesthetic appeal [3], it is especially remarkable that we still found the present associations that indicate other, more personal motivations for body modifications.

These findings corroborate previous research which highlighted the connection between the experience of sexual abuse and intimate piercings [30]. In the present study, we did not differentiate between pierced body parts, but the participant group who reported severe to extreme sexual abuse included the largest proportion of pierced (as well as of tattooed) individuals. Along these lines, the similar patterns observed for tattoos and piercings mirror previous reports of comparable motives [16].

In our study, we did not only observe the anticipated associations of physical and sexual abuse with body modifications, but we also found effects of emotional abuse and neglect. Therefore, piercings and tattoos might not only play a role in coping with negative experiences during which bodily autonomy was restricted or violated. Emotional abuse and neglect have also been highlighted as consequential early experiences which implicate mental distress later in life [45, 46]. Hence, survivors of these forms of childhood adversity might perceive the acquisition of body modifications as empowering, too. This hypothesis is supported by a previous study which found that especially individuals who also reported NSSI (an indicator of severe emotional pain which is common among survivors of abuse and neglect (e.g., [47]) cited emotional regulation as a reason for getting tattoos and piercings [24]. Likewise, female study participants with symptoms of unstable personality disorder (a specific pattern of personality pathology that has been linked with traumatic interpersonal, early experiences (e.g., [48]) differed from mentally healthy study participants regarding their motives for body modifications: They attached greater relevance to personal topics such as processing negative life events and coping [49].

These findings have several implications. On the one hand, it would be an unwarranted, overgeneralizing assumption to expect that people’s choices of tattoos and piercings are necessarily connected to stressful, early life events. In this study, neither presence nor severity of childhood abuse and neglect were perfectly correlated with body modifications. Previous research has also shown associations with more recent life events [25] and, beyond those, listed various other motivations (including more superficial ones) [16, 50, 51]. Especially as tattoos and piercings become part of Germany’s and other countries’ mainstream culture, the embellishment of the body can be assumed to be the primary goal of most people who get tattooed and/or pierced.

On the other hand, the present results indicate new, unconventional opportunities for creating access to psychosocial support, better screening, and potential starting points for interventions in psychotherapy.

First, it would be worth considering whether tattoo and piercing studios should be involved in population-based campaigns aimed at the mitigation of negative consequences of early life adversity. If clients disclose experiences of childhood abuse and neglect, the staff could pass on respective information material including contact details, providing low-threshold access to nearby clinics or counselling services. Clients could still decide for themselves whether to follow up on this offer.

Second, (mental) health care professionals should be aware of the potential significance of patients’ body modifications. If it is not already part of routine assessments, patients should be screened for a history of childhood abuse and neglect. In the following, a cautious exploration of their past experiences could contribute to emotional relief. It could also support the prevention of both mental and physical later-life sequelae of childhood adversity (e.g., through psychotherapy, psychoeducation, and adaptive health behaviors).

Third, the results suggested that patients’ tattoos and piercings could indicate topics of great significance to them (such as self-determination, or taking control). In the context of psychotherapy, clinicians could explore whether these are also currently important struggles in patients’ lives. The acknowledgement of tattoos and piercings as ways of self-expression could also facilitate conversations about individual ways of dealing with the past [52].

### Strengths and limitations

The large, representative population sample is a great strength of the present work, also because it precludes issues such as self-selection of participants (e.g., due to their special affinity for tattoos or piercings). We took a number of potentially confounding variables into account (gender, age, equivalized household income, and level of education). However, the study’s results need to be interpreted in the context of its limitations. First, the cross-sectional study design cautions against causal interpretations. Childhood abuse and neglect was assessed at a later stage in life and via self-report. However, self-reports of childhood abuse and neglect were deemed trustworthy [53]. Information regarding body modifications was also assessed in the form of a self-report and could have been more detailed: First, it was limited to tattoos and piercings and did not include other forms of body modification (such as scarification or transdermal and microdermal implants). Second, participants were not asked where their tattoos or piercings were located. There is evidence that the placement of a body modification is important with respect to its meaning for the wearer and with regard to its visibility/others’ reactions [4, 16]. Third, we did not collect detailed data about the total area or number of modifications, although both seem to be clinically relevant to distinguish fashion-motivated modifications from those used as emotional regulation or coping [8]. However, this aspect would be difficult to assess in quantitative surveys. Within the present context, assessments of current distress (e.g., symptoms of posttraumatic stress disorder) could have provided further clinically relevant insights. Regarding gender identity, the survey from which we drew our data forced a choice of the options woman or man. There was no option for nonbinary individuals and it did not differentiate between trans- and cisgender women and men either. Lastly, the current results are based on a German community sample. Therefore, they are only transferable to other cultures to a limited extent. This includes contexts in which body modifications are less common and viewed less favorably by the majority society because they are judged against a particular historical background, for example Japan, where tattoos still carry the stigma of criminal associations [54]. In strong contrast, some body modifications are very popular with other cultures and have great cultural significance for the individual and their community, for instance the piercing of the nose done by Indian women [54]. This limitation applies to most of the published research which heavily focuses on European or US-American surveys. Furthermore, as respective cultural factors are likely still relevant for migrated persons, it is a limitation of the present work that it did not differentiate between individuals of different origins and/or nationalities living in Germany.

## Conclusions

The present study adds to previous research by confirming positive and similar associations of tattoos and piercings with childhood abuse and neglect within a representative population sample. These relations did not just pertain to physical and sexual abuse, but also to early experiences of neglect and emotional forms of trauma. They were still observed in statistical models that controlled effects of potential socio-demographic confounders such as gender and age. Hence, for a substantial number of individuals who acquire body modifications, they could present a means of coping with previous adversity and be an expression of autonomy. These findings open up new avenues for support offers (involving tattoo artists and piercers) and screening (e.g., in primary care). Tattoos and piercings could also provide an impetus for therapeutic conversations about the significance of past experiences and about currently important themes.

## Data Availability

The dataset analyzed during the current study is available via the Open Science Framework: https://osf.io/3tqk4/?view_only=ffd4b97e8ecd4a538b472c9772da6fe5.
